# Supplementary material for: Topological phase singularities in atomically thin high-refractive-index materials
Source: Nat Commun. 2022 Apr 19;13:2049. doi: 10.1038/s41467-022-29716-4 (PMC9019097; doi:10.1038/s41467-022-29716-4)
Supplement: Supplementary file 1 — Supplementary Information [file 41467_2022_29716_MOESM1_ESM.docx]

**Supplementary Information**

**Topological phase singularities in atomically thin high-refractive-index materials**

Georgy Ermolaev,^1^ Kirill Voronin,^1^ Denis G. Baranov,^1^ Vasyl Kravets,^2^ Gleb Tselikov,^1^ Yury Stebunov,^3^ Dmitry Yakubovsky,^1^ Sergey Novikov,^1^ Andrey Vyshnevyy,^1^ Arslan Mazitov,^1, 4^ Ivan Kruglov,^1, 4^ Sergey Zhukov,^1^ Roman Romanov,^5^ Andrey M. Markeev,^1^ Aleksey Arsenin,^1, 6^ Kostya S. Novoselov,^3, 7, 8^ Alexander N. Grigorenko,^2^ and Valentyn Volkov^1, 9^

^1^Center for Photonics and 2D Materials, Moscow Institute of Physics and Technology, Dolgoprudny 141700, Russia.

^2^Department of Physics and Astronomy, University of Manchester, Manchester M13 9PL, UK.

^3^National Graphene Institute (NGI), University of Manchester, Manchester M13 9PL, UK.

^4^Dukhov Research Institute of Automatics (VNIIA), Moscow 127055, Russia.

^5^National Research Nuclear University MEPhI (Moscow Engineering Physics Institute), Moscow 115409, Russian Federation.

^6^GrapheneTek, Moscow 109004, Russia.

^7^Department of Materials Science and Engineering, National University of Singapore, Singapore 03-09 EA, Singapore.

^8^Chongqing 2D Materials Institute, Chongqing 400714, China.

^9^Xpanceo, Moscow 127495, Russia.

e-mail: [volkov.vs@mipt.ru](mailto:volkov.vs@mipt.ru)

**Table of Сontents**

**Supplementary Note 1: Phase singularities in high-refractive-index materials**

**Supplementary Note 2: Experimental evidences for topological protection of topological phase singularities**

**Supplementary Note 3: Derivation of the conditions for the observation of the zero-reflection points**

**Supplementary Note 4: Optical anisotropy in PdSe_2_**

**Supplementary Note 5: Determination of optical constants of PdSe_2_ from reflection and ellipsometry measurements**

**Supplementary Note 6: Ellipsometry measurement of PdSe_2_ in water medium**

**Supplementary Note 7: Biosensor characteristics (resolution, precision, and dynamic range)**

**Supplementary Note 8: Impossibility of existence of the higher-order topological charges on the** $\boldsymbol{r}_{\boldsymbol{p}}$ **and** $\boldsymbol{r}_{\boldsymbol{s}}$ **maps**

**Supplementary Note 9: Optical system with double topological charge**

**Supplementary Note 1: Phase singularities in high-refractive-index materials**

The effect of phase singularity is general and applicable not only to layered structures considered in the main part of the manuscript, but to a broader family of materials, for example, quasi-2D metals as well. Therefore, it allows to adjust the positions of topological points through the change of high-refractive-index materials. This concept is illustrated in Supplementary Figure 1 where topological points for different materials are collected.


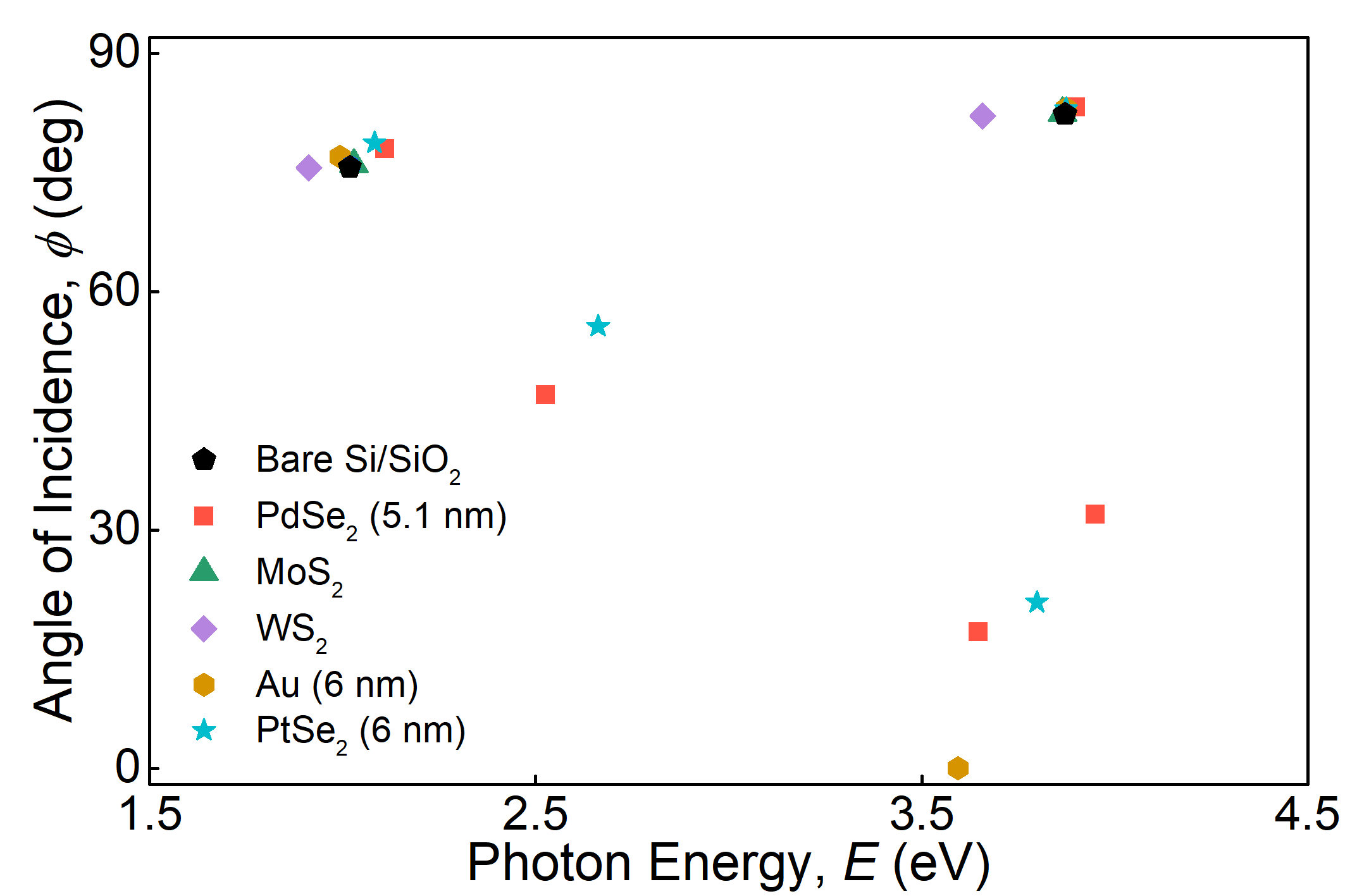


**Supplementary Figure 1. Abundance of phase singularities in high-refractive-index materials.** Calculated topological points using Supplementary Equation (4) for high-refractive-index materials. For comparison we also added topological points of bare substrate Si/SiO_2_. Optical constants for calculation were adopted from several reports.^1–5^

**Supplementary Note 2: Experimental evidences for topological protection of topological phase singularities**

In our work, the stability of topological phase singularities manifests itself in three ways: (i) in Figure 2a the reflection null was not achieved (around 4 eV) due to experimental non-idealities, while the phase topology preserved in Figure 2c; (ii) during the demonstration of the sensor operation; and (iii) in the theoretical modeling of the singular points annihilation. In the case of the sensor (Figure 4), changes in the refractive index of the solution do not lead to a disappearance of zero reflection point. The zero reflection point only shifts its position, through which we detect the change in the refractive index of the analyte. During the demonstration of annihilation of points (Figure 5a), the trajectories of motion of topological singularity points were drawn when the thickness of the film of a material with a high refractive index was changed. We showed that when the thickness changes, the points do not disappear but move in the space of frequencies and angles of incidence, however, two points with opposite charges can annihilate upon collision.

**Supplementary Note 3: Derivation of the conditions for the observation of the zero-reflection points**

In this work, ultrathin (with a thickness of about 5 nm or less) films of materials with a high refractive index are considered. In this case, we can assume that the wave phase acquired during the path through the film is small: $nk_{0}t\ll1$, where $k_{0}=\frac{\omega}{c}$, $\omega$ is the wave frequency, $c$ is the speed of light, $t$ is the thickness of the film and $n$ is the refractive index of the film material. This allows us in all analytical calculations to replace the film with a two-dimensional (2D) conductive layer. The effective 2D conductivity, $\sigma$ is related to the dielectric permittivity, $\varepsilon$, as: $\sigma=\frac{\varepsilon ct}{2i\lambda_{0}}$, where $\lambda_{0}$ is the free-space wavelength. We also use the normalized conductivity $\alpha=2\pi\sigma/c=\frac{\varepsilon k_{0}t}{2i}$, introduced for convenience. Due to this approximation, it is possible to obtain a relatively simple explicit expression for the dielectric constant that provides zero reflection at a given angle of incidence and wavelength of the incident light.

For this, we solve the standard linear system of equation for the reflection of the electromagnetic wave in the three-layer medium (see Figure 1a) but with the modified boundary condition for the magnetic field on the interface with 2D conductivity:

$$\boldsymbol{e}_{z}\times\left( \boldsymbol{H}_{t1}-\boldsymbol{H}_{t2} \right)=2\alpha\boldsymbol{E}_{t} (1)$$

where the subscript “t” in Eq. (1) means the in-plane components. Solving the linear system, we obtain

$$r_{s}=\frac{\left( q_{1z}-2\alpha\right)\left( q_{2z}\cos k_{2z}d-iq_{3z}\sin k_{2z}d \right)-q_{2z}\left( q_{3z}\cos k_{2z}d-iq_{2z}\sin k_{2z}d \right)}{\left( q_{1z}+2\alpha\right)\left( q_{2z}\cos k_{2z}d-iq_{3z}\sin k_{2z}d \right)+q_{2z}\left( q_{3z}\cos k_{2z}d-iq_{2z}\sin k_{2z}d \right)} (2)$$

Applying the condition $r_{s}=0$, we find $\alpha$ and dielectric permittivity of the film

$$\varepsilon_{s}=\frac{1}{k_{0}t}\left( iq_{1z}+q_{2z}\frac{q_{3z}-iq_{2z}\tan\left( k_{2z}d \right)}{q_{3z}\tan\left( k_{2z}d \right) +iq_{2z}} \right) (3)$$

Analogously, for the p-polarized light we obtain

$$\varepsilon_{p}=\frac{1}{k_{0}t}\left( \frac{i\varepsilon_{1}}{q_{1z}}+\frac{\varepsilon_{2}}{q_{2z}}\frac{\frac{\varepsilon_{3}}{q_{3z}}-\frac{i\varepsilon_{2}}{q_{2z}}\tan\left( k_{2z}d \right)}{q_{3z}\tan\left( k_{2z}d \right) +\frac{i\varepsilon_{2}}{q_{2z}}} \right) (4)$$

**Supplementary Note 4: Optical anisotropy in PdSe_2_**

PdSe_2_ is a naturally anisotropic material with a unique puckered pentagonal lattice structure (Figure 3a) with a point group *D*_2h_ and a space group *Pbca*. To get the qualitative insight into optical anisotropy, we theoretically calculated the real and imaginary parts of dielectric tensor of PdSe_2_ within the single-shot GW approach^6^ implemented in ViennaAb Initio Simulation Package (VASP).^7,8^ Core electrons and their effect on valence electrons were described in generalized gradient approximation (Perdew-Burke-Ernzerhof functional)^9,10^ using GW pseudopotentials with 18 and 24 valence electrons for Pd and Se respectively. Cutoff energy for plane waves basis set was 450 eV, and the first Brillouin zone was sampled with Γ-centered 4×4×3 mesh. The resulted optical constants are plotted in Supplementary Figure 2.


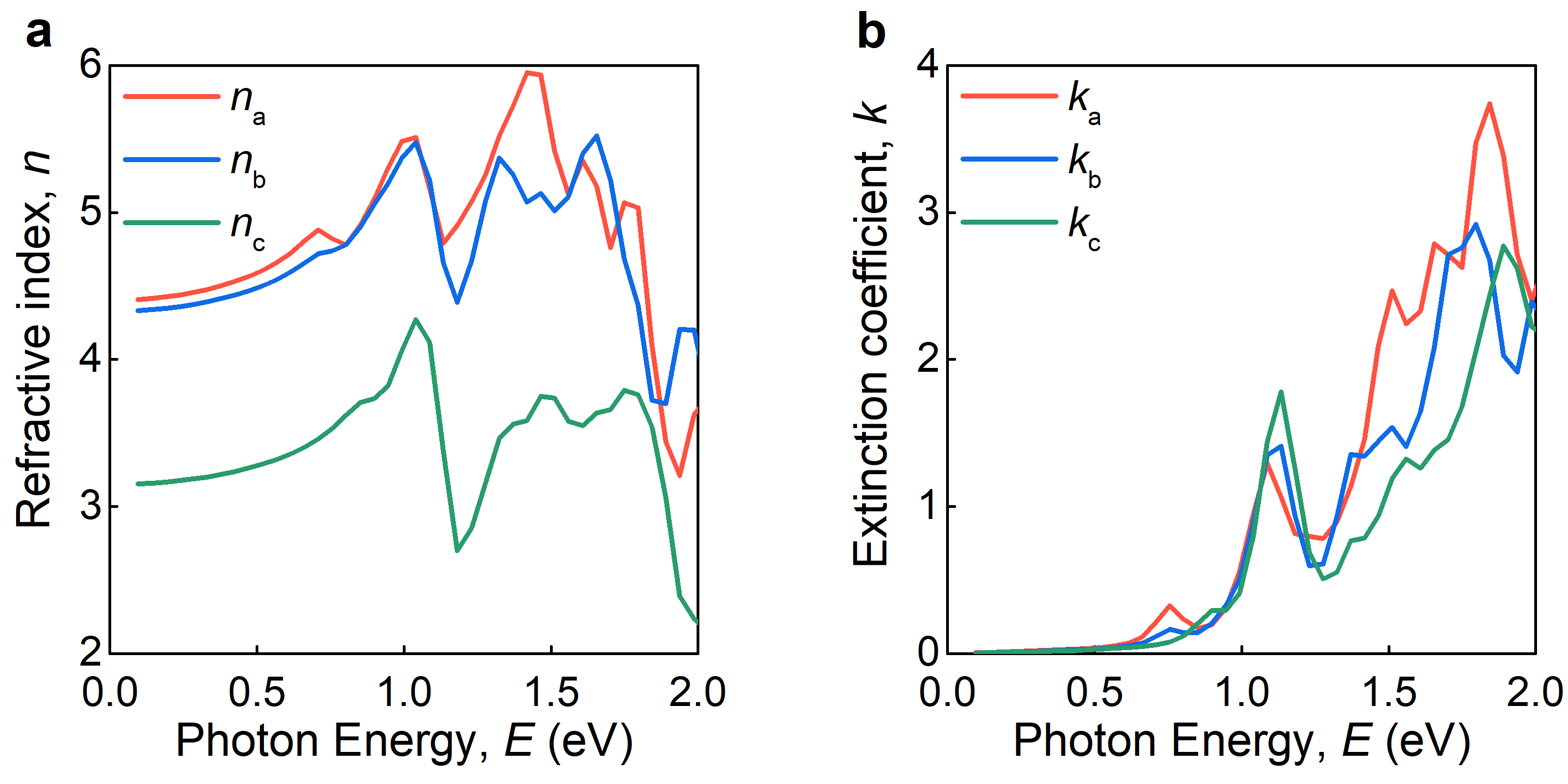


**Supplementary Figure 2. Theoretical optical anisotropy in PdSe_2_. a** Anisotropic refractive indices and **b** extinction coefficients along crystallographic axes a, b, and c.

To experimentally reveal PdSe_2_ optical anisotropy, we measured Mueller Matrix via imaging ellipsometer Accurion nanofilm_ep4. Mueller Matrix $\boldsymbol{M}$ relates the incident light polarization expressed in terms of Stokes parameters $S^{inc}= (S_{1}^{inc},S_{2}^{inc}, S_{3}^{inc},S_{4}^{inc})$ to the polarization of the light reflected from the sample $S^{ref}= (S_{1}^{ref},S_{2}^{ref}, S_{3}^{ref},S_{4}^{ref})$:^11^

$$S^{ref}=\boldsymbol{M}S^{inc} (5)$$

or in a detailed form:

$$\left\{ \begin{aligned} S_{1}^{ref}=m_{11}S_{1}^{\mathrm{inc}}+m_{12}S_{2}^{\mathrm{inc}}+m_{13}S_{3}^{\mathrm{inc}}+m_{14}S_{4}^{\mathrm{inc}} \\ S_{2}^{ref}=m_{21}S_{1}^{\mathrm{inc}}+m_{22}S_{2}^{\mathrm{inc}}+m_{23}S_{3}^{\mathrm{inc}}+m_{24}S_{4}^{\mathrm{inc}} \\ S_{3}^{ref}=m_{31}S_{1}^{\mathrm{inc}}+m_{32}S_{2}^{\mathrm{inc}}+m_{33}S_{3}^{\mathrm{inc}}+m_{34}S_{4}^{\mathrm{inc}} \\ S_{4}^{ref}=m_{41}S_{1}^{\mathrm{inc}}+m_{42}S_{2}^{\mathrm{inc}}+m_{43}S_{3}^{\mathrm{inc}}+m_{44}S_{4}^{\mathrm{inc}} \end{aligned} \right. (6)$$

where $m_{ij}$ are normalized to $m_{11}$, which is set to unity. In turn, Stokes parameters are observable quantities with the following definitions: the first Stokes parameter $S_{1}$ is the total intensity of light; $S_{2}$ characterizes linear horizontal (vertical) polarization; the parameter $S_{3}$ characterizes linear +45° polarization, and the parameter $S_{4}$ characterizes right and left circular polarization of the beam.^11^ Therefore, Stokes parameters describe light, while Mueller Matrix describes the sample of interest. More interestingly, off-diagonal elements of Mueller Matrix ($m_{13}$, $m_{14}$, $m_{23}$, $m_{24}$, $m_{31}$, $m_{32}$, $m_{41}$, $m_{42}$) indicate whether the sample has in-plane optical anisotropy (some off-diagonal elements are nonzero) or behaves isotropically (all off-diagonal elements are zero).^11^

In our device, we have access to 11 elements of Mueller Matrices shown in Supplementary Figure 3. Clearly, non-diagonal elements are non-zero, thus confirming in-plane optical anisotropy of PdSe_2_. Nevertheless, Mueller Matrices values differ from point to point. This difference originates, tentatively, from the random film growth during the CVD process. This assumption is in line with the value’s distribution following the Gaussian law (Supplementary Figure 4), showing that in-plane optical axis of PdSe_2_ orients randomly. More importantly, the average of off-diagonal elements of Mueller Matrices is zero which proves isotropic optical response of the PdSe_2_ sample at large scale.

Similar situation is also observed in optical microscope. During the rotation of polarizer, sample’s parts interchange colors as shown in Supplementary Figure 5 thereby validating local anisotropic response. Additionally, the Raman spectroscopy also demonstrates that the intensity of phonon modes differs for various polarizations of the excitation beam and again depends on position of the sample. Nevertheless, point measurements reveal expected dependence of the intensity $I\left( A_{g}^{1} \right)$ on angle $\alpha$ between the optical axis and the excitation beam polarization (Supplementary Figure 6):^12^

$$I\left( A_{g}^{1} \right)=\left( I_{a}\cos^{2} \left( \alpha\right)+I_{b}\sin^{2} \left( \alpha\right) \right)^{2} (7)$$

where $I_{a}$ and $I_{b}$ are eigenvalues of Raman tensor for $A_{g}^{1}$ phonon mode along crystallographic *a* and *b*-axes. These observations (ellipsometric, optical, and Raman) allow us to treat PdSe_2_ as an isotropic sample in the main part of the paper.


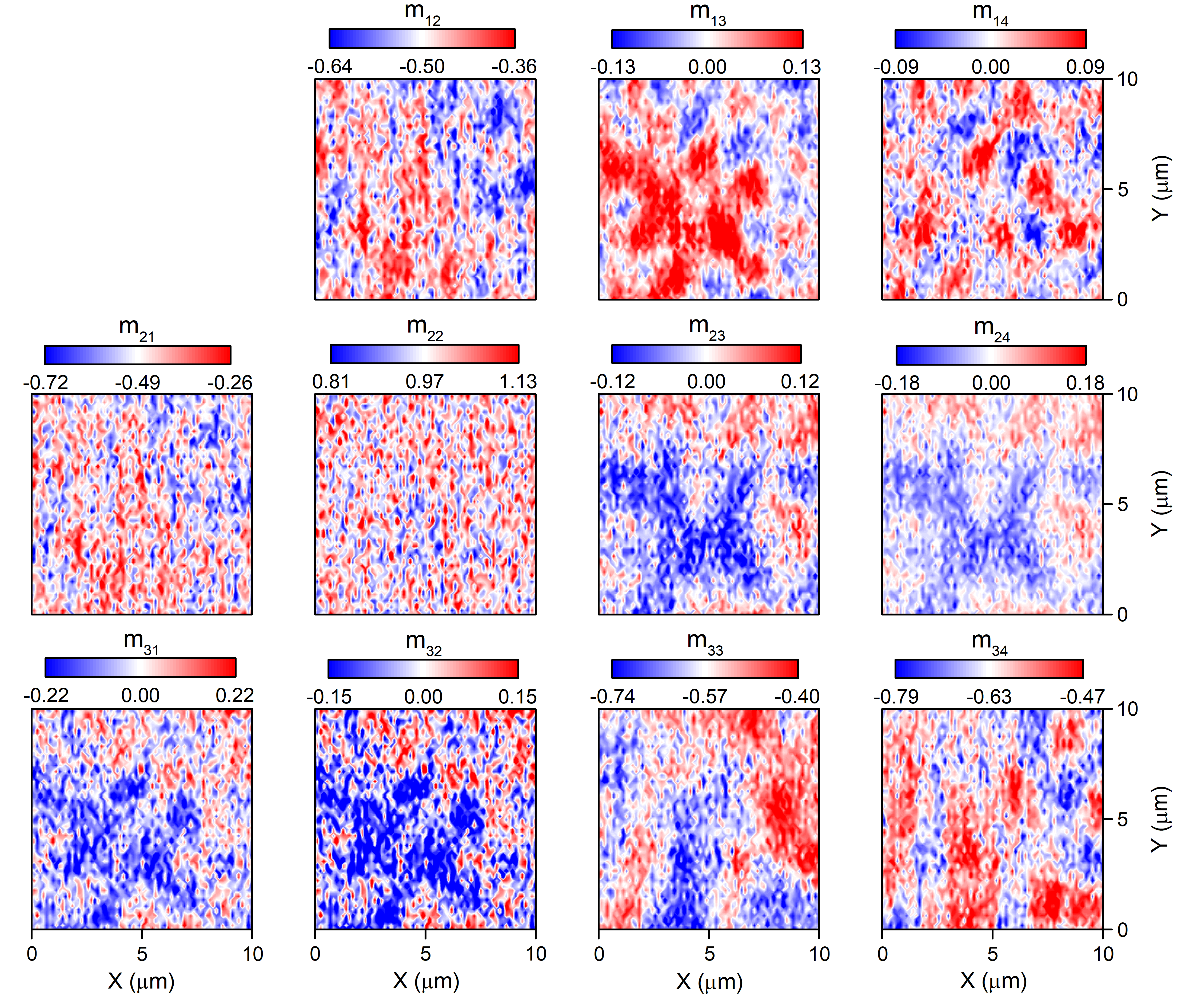


**Supplementary Figure 3. Anisotropy in PdSe_2_ imaged through Mueller Matrix.** Mueller Matrix recorded at 50° and $\lambda=$ 430 nm. Nonzero non-diagonal blocks of Mueller Matrix ($m_{13}$, $m_{14}$, $m_{23}$, $m_{24}$, $m_{31}$, $m_{32}$) shows that PdSe_2_ has in-plane anisotropy distributed randomly.


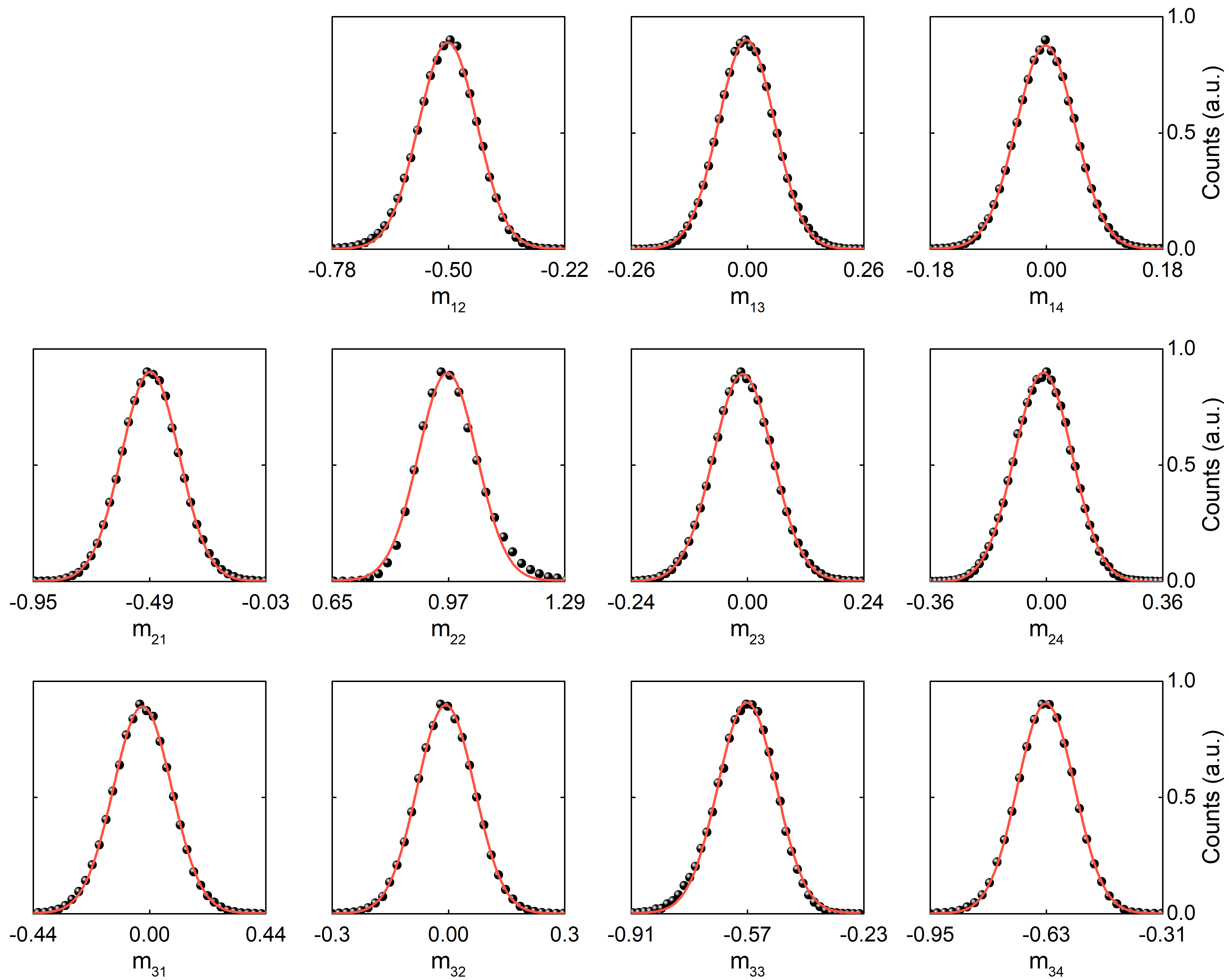


**Supplementary Figure 4. Distribution of Mueller Matrix values.** Relative frequency of Mueller Matrix values from Supplementary Figure 3 follows Gaussian distribution (red line) with zero average for non-diagonal blocks ($m_{13}$, $m_{14}$, $m_{23}$, $m_{24}$, $m_{31}$, $m_{32}$), thus validating isotropic response of PdSe_2_ at a macroscale.


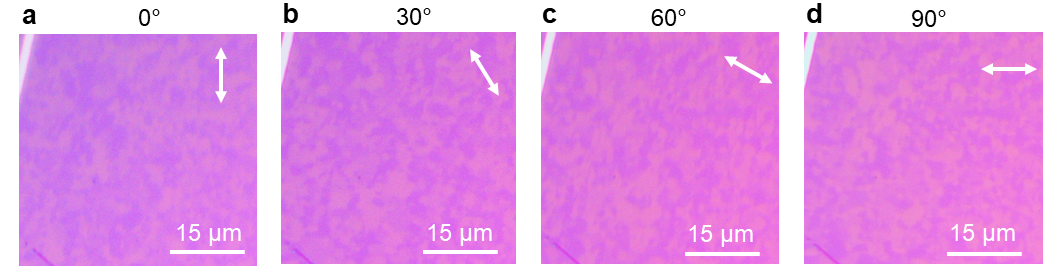


**Supplementary Figure 5. Optical images of PdSe_2_ at polarized illumination.** Macroscopic view of PdSe_2_ with polarizer at **a** 0°, **b** 30°, **c** 60°, and **d** 90°.


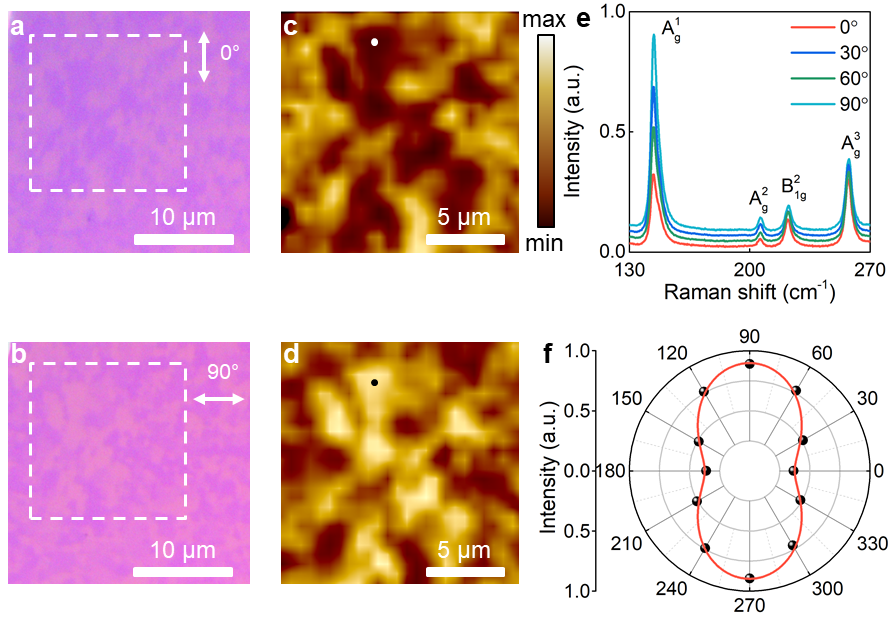


**Supplementary Figure 6. Polarized Raman spectroscopy of PdSe_2_. a-b** Optical images at Raman microscope at perpendicular orientations of polarizer. **c-d** Raman maps for intensity of $A_{g}^{1}$ taken at perpendicular polarizer angles from the sample area labeled by the dashed square in **(a-b)** panel, respectively. **e** Raman spectra for 4 polarizer angles taken at point denoted by white and black points in **(c-d)** panels. **f** The dependence of $A_{g}^{1}$ intensity on the angle of polarizer. The red line is a theoretical curve defined through Supplementary Equation (7).

**Supplementary Note 5: Determination of optical constants of PdSe_2_ from reflection and ellipsometry measurements**

To experimentally retrieve isotropic dielectric response of PdSe_2_ $\varepsilon_{\mathrm{PdSe}_{2}}$, we fitted ellipsometric spectra of $\Psi$ and $\Delta$ at 6 incidence angles, (45, 50, 55, 60, 65, 70°) and reflection spectrum at normal incidence presented in Supplementary Figure 7 a-b and c, respectively. We used 5 Tauc-Lorentz oscillators $\varepsilon_{\mathrm{TL}}^{'}+i\varepsilon_{\mathrm{TL}}^{''}$ model to describe excitonic response^4^ of PdSe_2_ and Drude oscillator $\varepsilon_{\mathrm{Drude}}$ to account metallic response from inherent doping revealed by XPS of valence band shown in Supplementary Figure 7d:

$$\varepsilon_{\mathrm{PdSe}_{2}}=\varepsilon_{\infty}+\varepsilon_{\mathrm{Drude}}+\sum_{j=1}^{5} \left( \varepsilon_{TL,j}^{'}+i\varepsilon_{TL,j}^{''} \right)=$$

$$=\varepsilon_{\infty}+\frac{-\hbar^{2}}{\varepsilon_{0}\rho\left( \tau E^{2}+i\hbar E \right)}+\sum_{j=1}^{5} \left( \frac{2}{\pi}v.p.\int_{E_{g}}^{\infty} \frac{\xi\varepsilon_{TL,j}^{''}\left( \xi\right)}{\xi^{2}-E^{2}}d\xi+iH_{E_{g}}\left( E \right)\frac{1}{E}\frac{A_{j}E_{0,j}C_{j}\left( E-E_{g} \right)^{2}}{\left( E^{2}-E_{0,j}^{2} \right)^{2}+C_{j}^{2}E^{2}} \right)(8)$$

where $\varepsilon_{\infty}$ is a contribution in the dielectric function of high energy interband transition, $E$ is photon energy, $\hbar$ is Planck’s constant, $\varepsilon_{0}$ is vacuum permittivity, $\rho$ is resistivity, $\tau$ is mean scattering time, $E_{g}$ is optical band gap, $H_{E_{g,}}\left( E \right)$ is Heaviside step function equal one if $E>E_{g,j}$ and zero otherwise, $A_{j}$, $C_{j}$, and $E_{0,j}$ are strength, broadening term, and peak central energy of *j*^th^ Tauc-Lorentz oscillator, respectively. Final parameters for PdSe_2_ optical model are $\varepsilon_{\infty}=$ 2.20 ± 0.02, $\rho=$ (0.0115 ± 0.0007) Ohm·cm, $\tau=$ (1.34 ± 0.07) fs, $E_{g}=$ (0.879 ± 0.004) eV with Tauc-Lorentz oscillator parameters collected in Supplementary Table 1.


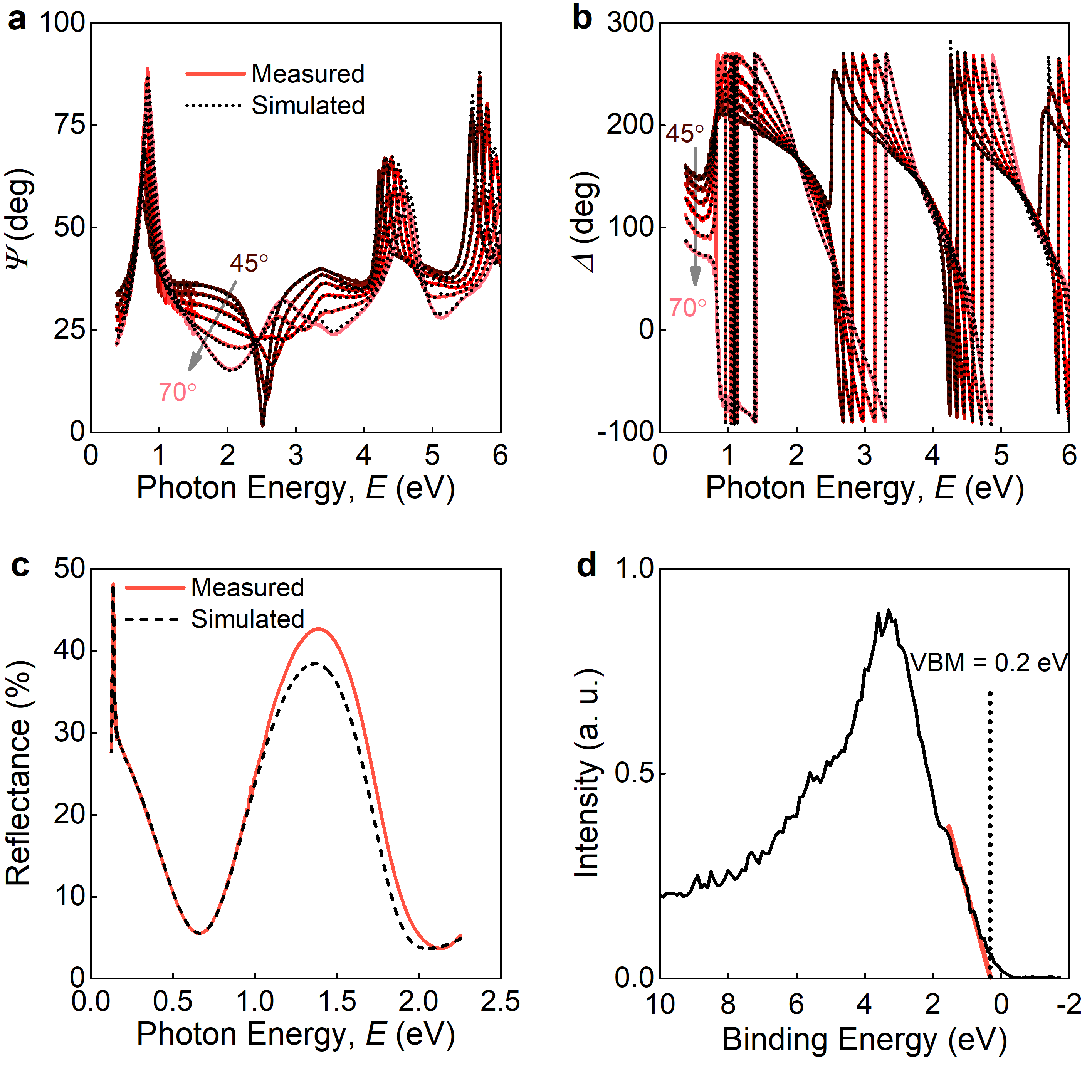


**Supplementary Figure 7. Determination of PdSe_2_ optical constants. a-b** Experimentally measured and simulated (based on optical model defined via Supplementary Equation (8)) ellipsometric parameters $\Psi$ (amplitude) and $\Delta$ (phase), and **c** normal incidence reflectance spectra for PdSe_2_(5.1 nm)/SiO_2_(280 nm)/Si. **d** Valence band revealed by XPS shows that maximum (VBM) is shifted from the Fermi level by 0.2 eV. It corresponds to p-type conductivity since PdSe_2_ bandgap is about 0.8 eV and undoped sample should have about 0.4 eV difference between Fermi level and VBM.^13^

**Supplementary Table 1.** Parameters of Tauc-Lorentz oscillators describing PdSe_2_ excitonic response.

| Tauc-Lorentz oscillator | *A* | *E*_0_ | *C* |
| --- | --- | --- | --- |
|  | eV | eV | eV |
| #1 | 54.8 ± 0.6 | 2.027 ± 0.002 | 1.172 ± 0.005 |
| #2 | 4.72 ± 0.15 | 3.090 ± 0.005 | 0.789 ± 0.015 |
| #3 | 6.4 ± 0.2 | 3.766 ± 0.005 | 0.958 ± 0.017 |
| #4 | 0.377 ± 0.012 | 5.048 ± 0.005 | 0.163 ± 0.014 |
| #5 | 35 ± 1 | 6.89 ± 0.04 | 9.54 ± 0.29 |

**Supplementary Note 6: Ellipsometry measurement of PdSe_2_ in water medium**

For PdSe_2_/SiO_2_/Si to be a reliable sensor, PdSe_2_ should preserve its properties (in particular, optical constants from Figure 3k) when contacted with water solution. To confirm PdSe_2_ water stability, we measured ellipsometric maps in the vicinity of topological zero of the system. Evidently, measured and simulated (based on dielectric function from Figure 3k) spectra in Supplementary Figure 8 are in close agreement thereby validating that water has no effect on PdSe_2_ dielectric function. Hence, we can safely use PdSe_2_ as a liquid refractive index sensor.


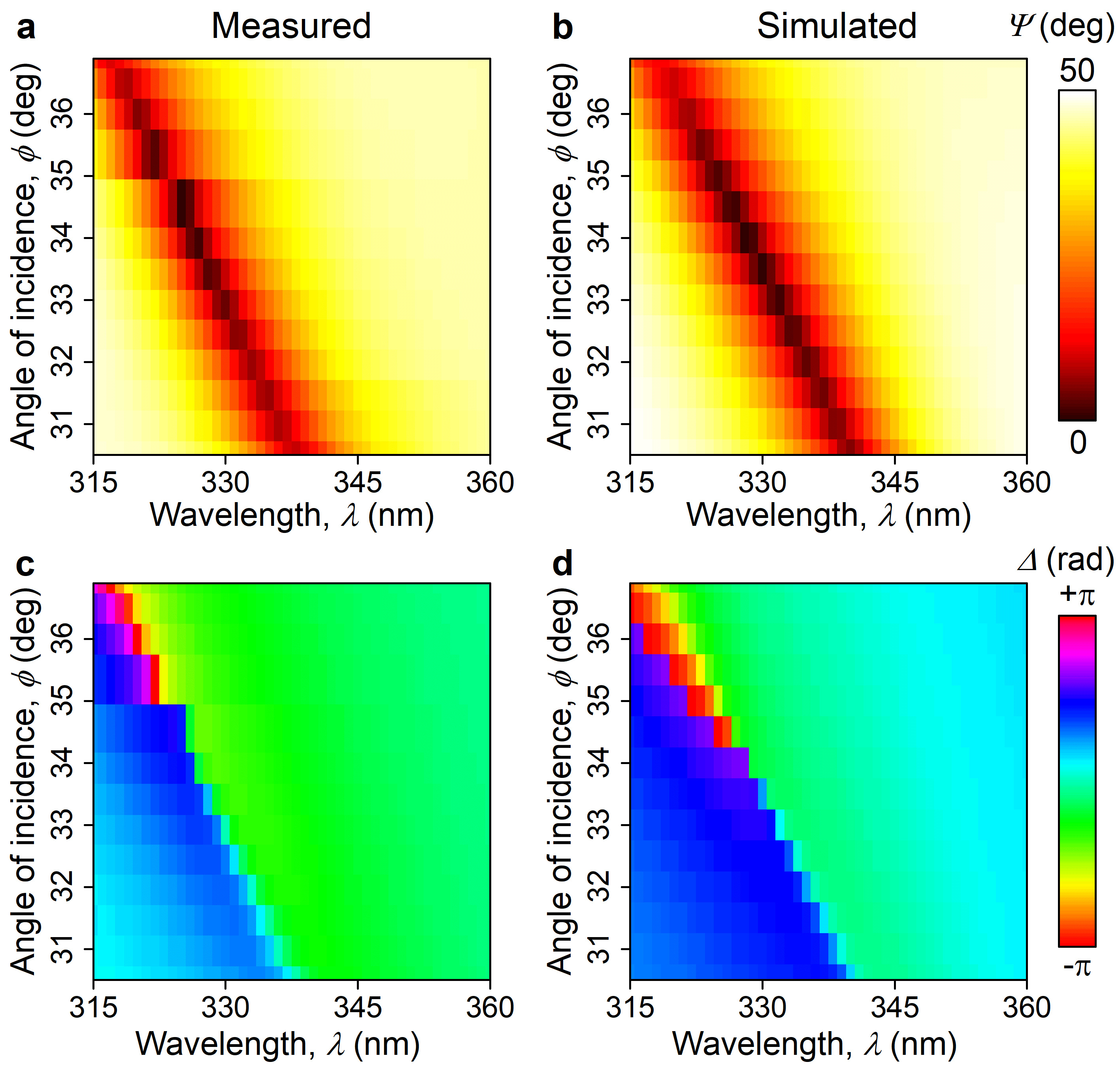


**Supplementary Figure 8. Topological point of PdSe_2_ in water. a-b** Experimental and **c-d** simulated ellipsometric parameters $\Psi$ (amplitude) and $\Delta$ (phase) for the system water/PdSe_2_/SiO_2_/Si.

**Supplementary Note 7: Biosensor characteristics (resolution, precision, and dynamic range)**

i) Resolution (minimum detectable increment):

In our device, the minimum detectable increment was Δn = 0.00125. However, we believe that this result can be further increased by the change of the detection system since commercial ellipsometers (used in the study) are designed for optical characterization but not for sensing. As a result, ellipsometers have a high phase noise level. In contrast, one can use an advanced photoacoustic modulator-based scheme of phase detection,^14^ which can detect the phase change of 5·10^-3^ deg. Using the improved phase-detection scheme, our sensor resolution would improve to Δn = 0.7·10^-7^.

ii) Precision (repeatability of results):

We repeated our sensor measurements after 9 months, and phase sensitivity was fully reproduced: in January 2021, we had 7.5·10^4^ deg/RIU (Figure 4), while in October 2021, we obtained 7.6·10^4^ deg/RIU (Supplementary Figure 9). At the same time, the position of phase singularity slightly changed from *λ* = 325.9 nm and $\phi$ = 49.4° at January to λ = 327.3 nm and $\phi$ = 48.4° at October, corresponding to Δλ = 1.4 nm and Δ$\phi$ = 1°. Hence, our sensor precision (repeatability of results) is high thanks to topological protection but requires recalibration after a month of usage.

iii) Dynamic range (range of refractive indices supported by the device):

Traditionally, optical biosensors work in water or buffer solutions because these media provide for biomolecules optimal parameters (pH), which leads to effective biomolecules adsorption to the sensor surface. As a result, we also tested our biosensor in the range 1.35 - 1.36, which is our experimentally proved dynamical range (range of refractive indices supported by the device).

Additionally, we want to emphasize that our sensor is mostly designed for the registration of small refractive index increments since for small increments our device has the highest sensitivity owing to the rapid phase change near the topological point. Nonetheless, for a higher refractive index increment (Δn > 0.01), one can use the change in spectral position of the topological point (Figure 4c or Supplementary Figure 9c). To sum up, the proposed sensor can potentially be used in any range of refractive indices, but to exploit the full advantage of topological phase singularity, one should leverage the sensor close to the calibrated solution (in our case, water).

The final remark is that one can simultaneously use several singularity points at the same time (Figure 2a-d) to increase the sensitivity and robustness of the sensor. For example, register the phase change in the vicinity of two singularity points at the same time (Figure 2a-d) to increase sensor sensitivity and robustness: one topological point results in higher sensitivity for small changes, whereas another topological point yields better response for higher increment.


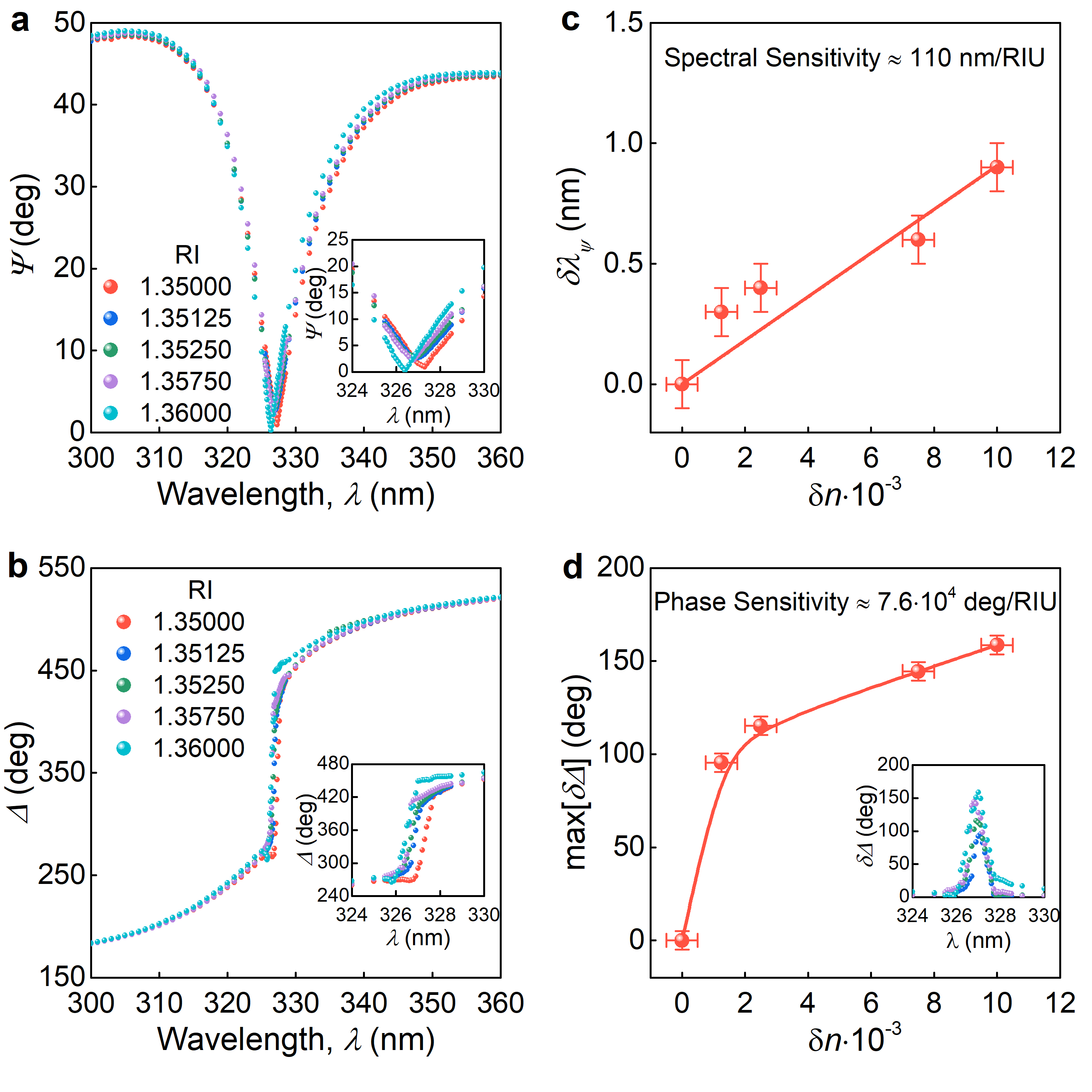


**Supplementary Figure 9. Topological point of PdSe_2_ in water after 9 months. a-b** Experimental and **c-d** simulated ellipsometric parameters $\Psi$ (amplitude) and $\Delta$ (phase) for the system water/PdSe_2_/SiO_2_/Si. Clearly, the result is close to Figure 4 owing to the topological nature of the proposed sensor.

**Supplementary Note 8: Impossibility of existence of the higher-order topological charges on the** $\boldsymbol{r}_{\boldsymbol{p}}$ **and** $\boldsymbol{r}_{\boldsymbol{s}}$ **maps**

1. *Reflection coefficient near the topological point*

Without loss of generality, we will start with the expression for the reflection coefficient for p- polarization:

$$r_{p}=\frac{\frac{\varepsilon_{2}q_{1z}}{\varepsilon_{1}q_{2z}}\left( \frac{q_{2z}}{\varepsilon_{2}}\cos k_{2z}d-i\frac{q_{3z}}{\varepsilon_{3}}\sin k_{2z}d \right)-\left( 1-2\alpha\frac{q_{1z}}{\varepsilon_{1}} \right)\left( \frac{q_{3z}}{\varepsilon_{3}}\cos k_{2z}d-i\frac{q_{2z}}{\varepsilon_{2}}\sin k_{2z}d \right)}{\frac{\varepsilon_{2}q_{1z}}{\varepsilon_{1}q_{2z}}\left( \frac{q_{2z}}{\varepsilon_{2}}\cos k_{2z}d-i\frac{q_{3z}}{\varepsilon_{3}}\sin k_{2z}d \right)+\left( 1+2\alpha\frac{q_{1z}}{\varepsilon_{1}} \right)\left( \frac{q_{3z}}{\varepsilon_{3}}\cos k_{2z}d-i\frac{q_{2z}}{\varepsilon_{2}}\sin k_{2z}d \right)} (9)$$

Near a zero-reflection point ($r_{p}(\lambda_{0},\theta_{0})=0$), the reflection coefficient could be represented as

$$r_{p}=\frac{\partial r_{p}}{\partial\lambda}\Delta\lambda+\frac{\partial r_{p}}{\partial\theta}\Delta\theta\mathcal{+O}\left( \sqrt{\frac{\Delta\lambda^{2}}{\lambda_{0}^{2}}+\frac{\Delta\theta^{2}}{\theta_{0}^{2}}} \right) (10)$$

where $\Delta\lambda= \lambda- \lambda_{0}$, $\Delta\theta= \theta-\theta_{0}$, and $\frac{\partial r_{p}}{\partial\lambda}$ and $\frac{\partial r_{p}}{\partial\theta}$ are calculated in $(\lambda_{0},\theta_{0})$ point. Rewriting $r_{p} as$:

$$\left( \text{Re}\left[ \frac{\partial r_{p}}{\partial\lambda} \right]\Delta\lambda+\text{Re}\left[ \frac{\partial r_{p}}{\partial\theta} \right]\Delta\theta\right)+i\left( \text{Im}\left[ \frac{\partial r_{p}}{\partial\lambda} \right]\Delta\lambda+\text{Im}\left[ \frac{\partial r_{p}}{\partial\theta} \right]\Delta\theta\right)=\Delta x+i\Delta y=\Delta z, (11)$$

we see that the map $(\Delta\lambda, \Delta\theta) \to(\Delta x, \Delta y)$ is linear, and while it is not degenerate, it transforms a circle into an ellipse with the same center. Therefore, $|C|=1$.

1. *Higher-order charges conditions*

To achieve a higher-order singularity, we need that $r_{p}=\Delta z^{\left| C \right|}\mathcal{+O}\left( \Delta z^{\left| C \right|+1} \right)$, for some $\Delta z=\left( A\Delta\lambda+B\Delta\theta\right)+i\left( C\Delta\lambda+D\Delta\theta\right)$. Therefore, at least (necessary but not sufficient) the following conditions must be satisfied:

$\frac{\partial r_{p}}{\partial\lambda}\left( \lambda_{0},\theta_{0} \right)=0$ (12)

$\frac{\partial r_{p}}{\partial\theta}\left( \lambda_{0},\theta_{0} \right)=0$ (13)

Equation $r_{p}\left( \lambda_{0},\theta_{0} \right)=0$ gives us the condition for the $\alpha$: $\alpha=f_{1}\left( \lambda_{0},\theta_{0} \right)$, Eqs. (12,13) give the second condition: $\alpha=f_{2}\left( \lambda_{0},\theta_{0} \right)$. As both conditions should be fulfilled simultaneously, $f_{1}\left( \lambda_{0},\theta_{0} \right)=f_{2}\left( \lambda_{0},\theta_{0} \right)$, which means that only several separate points could have non-unitary charge.

Let us now switch to the second representation of the topology and consider the intersection of the material curve with the zero-reflection surface in the point corresponding to $(\lambda_{0},\theta_{0})$. Eq. (13) with the condition $\alpha=f_{1}\left( \lambda_{0},\theta_{0} \right)=f_{2}\left( \lambda_{0},\theta_{0} \right)$ fixes also the derivative of $\alpha$ with respect to the wavelength, which defines the direction of the material curve. Moreover, an infinitesimal change of the material curve’s direction leads to the appearance of the linear terms and disappearance of the $|C|>1$ point. Indeed, one can show that

$$r_{p}=\left( \text{Re}\left[ \frac{\partial r_{p}}{\partial\lambda} \right]\Delta\lambda+\text{Re}\left[ \frac{\partial^{2}r_{p}}{\partial\lambda\partial\theta} \right]\Delta\lambda\Delta\theta+\text{Re}\left[ \frac{\partial^{2}r_{p}}{\partial\theta^{2}} \right]\Delta\theta^{2} \right)+i\left( \text{Im}\left[ \frac{\partial r_{p}}{\partial\lambda} \right]\Delta\lambda+\text{Im}\left[ \frac{\partial^{2}r_{p}}{\partial\lambda\partial\theta} \right]\Delta\lambda\Delta\theta+\text{Im}\left[ \frac{\partial^{2}r_{p}}{\partial\theta^{2}} \right]\Delta\theta^{2} \right) (14)$$

corresponds to $|C|<2$.

For each arrangement of the curve and surface one can find an infinitesimal rotation of the curve which will not lead to the appearance of additional intersections. The rest point will have $|C|<2$, which means that an initial point also has $|C|<2$, because of the topological charge conservation.

**Supplementary Note 9: Optical system with double topological charge**

To theoretically realize zero with double topological charge $C=$ +2, we use thin film incorporated in the structure dielectric ($\varepsilon=$ 5)/thin film (5 nm)/SiO_2_ (300 nm)/Si and with optical constants defined via Lorentz oscillator:

$$\varepsilon=\varepsilon_{\infty}+\frac{A}{\omega_{0}^{2}-\omega^{2}-i\omega\gamma} (15)$$

with $\varepsilon_{\infty}=$ 10*i*; $A=$ 10^-4^ nm^-2^; $\lambda_{0}=$ 374.6 nm, $\omega_{0}=$1$/\lambda_{0}$; $\gamma=$0.00324 nm^-1^. The resulting topological zero is plotted in Figure 5c of the main text.

Another approach is to use in-plane anisotropy, which greatly helps in the engineering of double topological charge when optical axis of the thin film oriented along with in-plane component of wave vector. It allows independent control of p- and s-polarization response through optical constants of thin film. As an example, the system anisotropic film (5 nm)/SiO_2_ (280 nm)/Si yields double topological charge (Figure 5d) with the following Lorentz parameters for anisotropic film: for optical axis parallel to in-plane wave vector $\varepsilon_{\infty}=$ 20; $A=$ 10^-4^ nm^-2^; $\lambda_{0}=$ 501.55 nm, $\omega_{0}=$1$/\lambda_{0}$; $\gamma=$0.00021 nm^-1^; for optical axis perpendicular to in-plane wave vector $\varepsilon_{\infty}=$ 1; $A=$ 10^-5^ nm^-2^; $\lambda_{0}=$ 420 nm, $\omega_{0}=$1$/\lambda_{0}$; $\gamma=$0.0002 nm^-1^.

**SUPPLEMENTARY REFERENCES**

1. Wang, Y. *et al.* Atomically Thin Noble Metal Dichalcogenides for Phase-Regulated Meta-optics. *Nano Lett.* **20**, 7811–7818 (2020).

2. Kravets, V. G. *et al.* Spectroscopic ellipsometry of graphene and an exciton-shifted van Hove peak in absorption. *Phys. Rev. B - Condens. Matter Mater. Phys.* **81**, 1–6 (2010).

3. Ermolaev, G. A. *et al.* Broadband optical properties of monolayer and bulk MoS2. *npj 2D Mater. Appl.* **4**, 1–6 (2020).

4. Ermolaev, G. A., Yakubovsky, D. I., Stebunov, Y. V., Arsenin, A. V. & Volkov, V. S. Spectral ellipsometry of monolayer transition metal dichalcogenides: Analysis of excitonic peaks in dispersion. *J. Vac. Sci. Technol. B* **38**, 014002 (2020).

5. Tatarkin, D. E. *et al.* Surface-enhanced Raman spectroscopy on hybrid Graphene/Gold substrates near the percolation threshold. *Nanomaterials* **10**, (2020).

6. Shishkin, M. & Kresse, G. Implementation and performance of the frequency-dependent GW method within the PAW framework. *Phys. Rev. B - Condens. Matter Mater. Phys.* **74**, 1–13 (2006).

7. Kresse, G. & Furthmüller, J. Efficient iterative schemes for ab initio total-energy calculations using a plane-wave basis set. *Phys. Rev. B* **54**, 11169–11186 (1996).

8. Kresse, G. & Joubert, D. From ultrasoft pseudopotentials to the projector augmented-wave method. *Phys. Rev. B* **59**, 1758–1775 (1999).

9. Blöchl, P. E. Projector augmented-wave method. *Phys. Rev. B* **50**, 17953–17979 (1994).

10. Perdew, J. P., Burke, K. & Ernzerhof, M. Generalized gradient approximation made simple. *Phys. Rev. Lett.* **77**, 3865–3868 (1996).

11. Goldstein, D. H. *Polarized Light*. (CRC Press, 2017). doi:10.1201/b10436.

12. Yu, J. *et al.* Direct Observation of the Linear Dichroism Transition in Two-Dimensional Palladium Diselenide. *Nano Lett.* **20**, 1172–1182 (2020).

13. Lu, L.-S. *et al.* Layer-Dependent and In-Plane Anisotropic Properties of Low-Temperature Synthesized Few-Layer PdSe 2 Single Crystals. *ACS Nano* **14**, 4963–4972 (2020).

14. Kabashin, A. V., Patskovsky, S. & Grigorenko, A. N. Phase and amplitude sensitivities in surface plasmon resonance bio and chemical sensing. *Opt. Express* **17**, 21191 (2009).
